# Molecular characterization of *Cryptosporidium* and *Giardia* occurring in natural water bodies in Poland

**DOI:** 10.1007/s00436-014-4234-9

**Published:** 2014-12-05

**Authors:** Małgorzata Adamska

**Affiliations:** Department of Genetics, Szczecin University, Felczaka 3c, 71-412 Szczecin, Poland

**Keywords:** *Cryptosporidium parvum*, *Giardia intestinalis*, Natural water bodies, Genotyping, Phylogenetic analysis, Contamination source

## Abstract

*Cryptosporidium* and *Giardia* protozoa are zoonotic parasites that cause human gastroenteritis and can be transmitted to human through the fecal-oral route and water or food. Several species belong to these genera and their resistant forms occur in water, but only some of them are infectious to human. Health risk depends on the occurrence of infectious *Cryptosporidium* and *Giardia* species and genotypes in water, and only molecular techniques allow detecting them, as well as enable to identify the contamination source. In this work, genotyping and phylogenetic analysis have been performed on the basis of *18S rDNA* and *ß*-*giardin* genes sequences of *Cryptosporidium* and *Giardia*, respectively, in order to provide the molecular characterization of these parasites detected earlier in five natural water bodies in Poland and to track possible sources of their (oo)cysts in water. Genotyping revealed a high similarity (over 99 up to 100 %) of analyzed sequences to cattle genotype of *C. parvum* isolated from cattle and human and to *G. intestinalis* assemblage B isolated from human. The sequences obtained by others originated from patients with clinical symptoms of cryptosporidiosis or giardiasis and/or with the infection confirmed by different methods. The contamination of three examined lakes is probably human-originated, while the sources of contamination of two remaining lakes are wild and domestic animals. Obtained phylogenetic trees support suggestions of other authors that the bovine genotype of *C. parvum* should be a separate species, as well as A and B assemblages of *G. intestinalis*.

## Introduction

*Cryptosporidium* is a genus including at least 25 species and 60 genotypes, however, not all of them cause the same level of risk to human (2010; Rossle and Latif 2013; Šlapeta 2013). *Cryptosporidium hominis* and *Cryptosporidium parvum* are responsible for over 90 % of cryptosporidiosis cases in human (Rossle and Latif 2013). Moreover, other species have been detected in immunocompetent humans (Rossle and Latif 2013), so there is a threat of zoonotic transmission in the environment. The genotyping techniques can differentiate *Cryptosporidium* species to detect those infecting human, as well as to track sources of contamination, because most *Cryptosporidium* species and genotypes are host specific (Xiao and Feng 2008). The genus *Giardia* can be differentiated into at least six species based on their DNA polymorphism (Ryan and Cacciò 2013) and only *Giardia intestinalis* infects humans. This one is a complex species that contains eight distinct genotypes (assemblages) and only two of them (A and B) are associated with human infection and have also been found in different species of other mammals (Dado et al. 2012; Ryan and Cacciò 2013). The genotypes cannot be distinguished on the basis of host origin or parasite morphology (Xiao and Fayer 2008), so genotyping is the most useful method for identification of the assemblages infective for human.

The presence of *Cryptosporidium* spp. oocysts and *Giardia* spp. cysts in water is an increasing problem throughout the world, and these protozoa are causes of widespread gastrointestinal diseases and morbidity in human and animals (Hajdušek et al. 2004; Xiao and Fayer 2008; Ruecker et al. 2012; Rossle and Latif 2013; Ryan and Cacciò 2013). The precise identification of a parasite at the species and/or genotype level is of a great importance for various aspects of human and veterinary parasitology, including taxonomy, diagnosis, and treatment (Ruecker et al. 2012), and genotyping is necessary to evaluate the risk of infection for both human and animals. For these reasons, genotyping and phylogenetic analysis have been performed in order to provide the molecular characterization of *Cryptosporidium* and *Giardia* detected earlier (Adamska et al. 2014) in natural water bodies in Poland.

## Materials and methods

The sequences of *Cryptosporidium* 18S rDNA gene and *Giardia ß*-*giardin* gene analyzed in this study were obtained earlier from the water samples collected from 36 natural water bodies in north-western Poland (Adamska et al. 2014). Afterwards, they were aligned with published homology sequences with Mega 5.10 software by using ClustalW (Tamura et al. 2011), and they have been deposited in the GenBank database under accession numbers KC748017-23. The sequences were also used for phylogenetic analysis with sequences obtained by others (AB441688, AF093490, AF112572, AF115377, AJ493074, AJ493079, AJ493084, AJ493531, AJ849462, AY120901, AY268583, AY458613, DQ182559, DQ523510, DQ898159, DQ898160, FJ262725, GQ227705, JQ250803, KC608024 for *Cryptosporidium* and FJ009207, DQ090530, JQ684217, DQ116622, DQ116615, DQ116606, EU189373, EU189369, EU216429, AY072724, JQ684209, JQ247032, AY655702, HQ538712, DQ648780, AY258618 for *Giardia*). Before the phylogenetic analysis, the ends of the alignments were trimmed in order to form blunt ends on all the sequences in the alignment. The final alignments covered 736 nucleotides corresponding to nucleotide positions 18 to 754 of *C. parvum* with GenBank accession numbers KC748017-19 and 1 to 328 of *G. intestinalis* with GenBank accession numbers KC748020-23. Phylogenetic trees were constructed with Mega 5.10 software by using the maximum likelihood method and the Kimura two-parameter model with 1000 bootstrap sampling (Tamura et al. 2011). *Cryptosporidium muris* and *Giardia muris* sequences (JX948127 and AY258618, respectively) were used as the outgroups in order to root the trees.

## Results

### Genotyping

Sequence analysis of three *Cryptosporidium* 18S rDNA PCR products revealed the presence of three variants. One of the sequences (KC748019) was identical to the bovine genotype BOH6 (AF093490) of *C. parvum* and other sequences originated from this species, isolated from: cattle (AB441687, EF611871, AY204237, AY204238, HQ009805, JX416362, JX298604, AB513870-81, AB513865-68, AF108864, JN120853), sheep (JN247404), deer (AF093494), alpaca (EF375894), rodents (HQ651731, GQ121019), and a fox (HQ822132), as well as in human patients (AJ849461, EU331237, EU331238, EU331241, GQ983351, GQ983355, JQ413434, HQ332160, AB434889, DQ067566, AJ493201, AB089290, AJ493544, AJ493547, DQ523504) from different countries. Two remaining sequences (KC748017 and KC748018) were unique and their similarity to AF093490 and the sequences mentioned above was 99.87 %.

According to the region of β-giardin gene PCR sequence analysis, three variants of *G. intestinalis* assemblage B were detected in all four positive samples. The similarity of these sequences to assemblage B sequence (HM165210) isolated from stool of patient and to other sequences obtained from humans from different countries (HM165210, DQ090522, DQ090523, DQ090525, DQ090527, DQ923579, AY072726, JQ684210, JQ684212, AY258616, AB618785, AY072727) was 100 % (KC748020 and KC748021) and 99 % (KC748022 and KC748023).

### Phylogenetic analysis

**Fig. 1:**
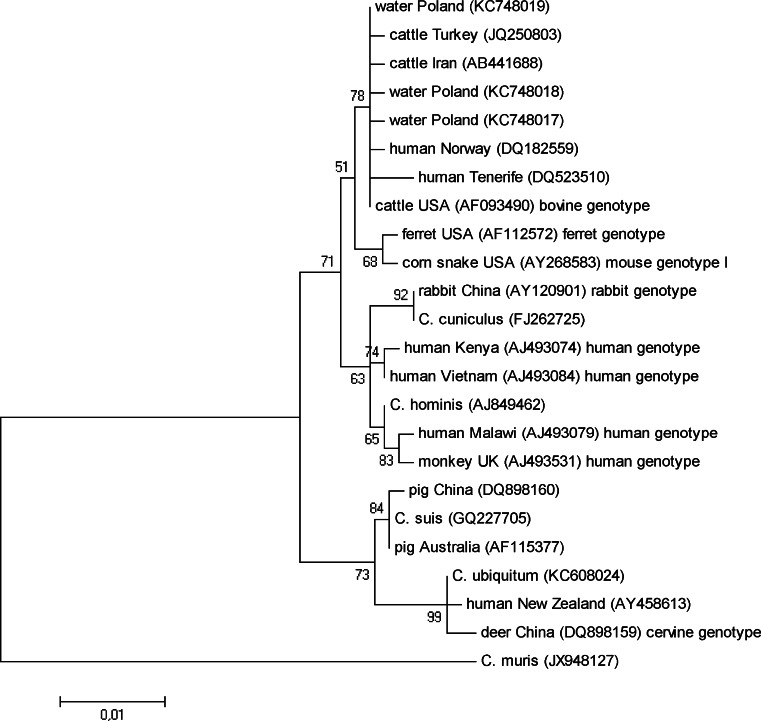
The phylogenetic tree for *Cryptosporidium*

According to obtained phylogenetic tree (Fig. 1), the *Cryptosporidium* isolates analyzed in this study cluster together with the strain described as *C. parvum* bovine genotype and other *C. parvum* strains originated from cattle and humans. This clad forms a group to the sequences representing *C. parvum* ferret genotype and *C. parvum* mouse genotype I. The second large group contains the sequences originated from humans and a monkey described as *C. parvum* human genotype, the strain of *C. hominis*, as well as the sequence described as *C. parvum* rabbit genotype and the strain of *Cryptosporidium cuniculus*. The two large groups described above cluster together, as opposed to the third group containing a clad that consists of *Cryptosporidium suis* strain and two *Cryptosporidium* strains originated from pigs, and a clad containing *Cryptosporidium ubiquitum* strain, the strain described as *C. parvum* cervine genotype and the strain originated from a human.

**Fig. 2:**
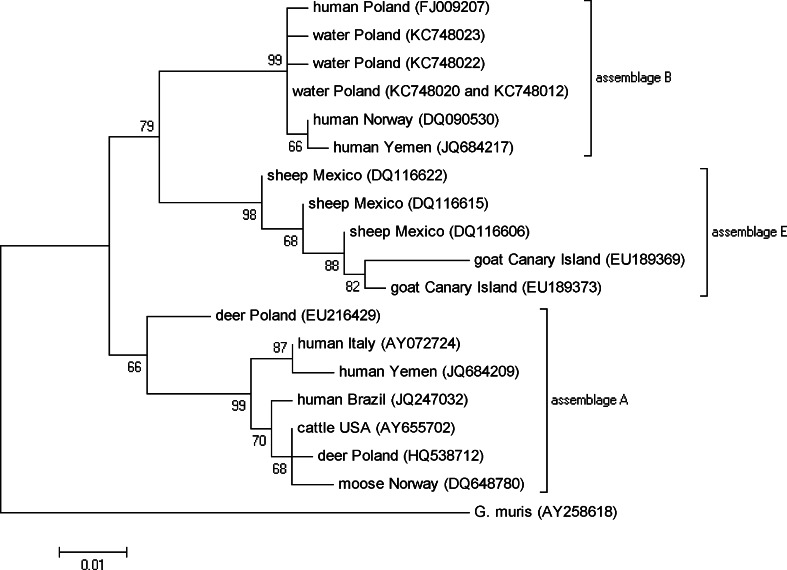
The phylogenetic tree for *Giardia*

The phylogenetic tree for *Giardia* (Fig. 2) shows that the sequences analyzed in this study cluster with sequences originated from human representing assemblage B of *G. intestinalis*. The clade consisting of these sequences clusters with the clade containing assemblage E sequences obtained from sheep and goat isolates. The rest of sequences used for tree construction comprise the big clade containing assemblage A sequences obtained from human and ruminants.

## Discussion

Many water samples have been shown to contain *Cryptosopridium* oocysts, but not all the species and genotypes identified in water are health-threatening for human (Xiao and Feng 2008; Ruecker et al. 2012; Rossle and Latif 2013; Šlapeta 2013). In this study, the DNA of *Cryptosporidium* oocysts from three lakes was analyzed, and in all the samples the presence of the bovine genotype of *C. parvum* was revealed. *C. parvum* is a multispecies complex containing several genotypes and some of them have been named as separate species, e.g., *C. parvum* human genotype was named *C. hominis* (Šlapeta 2006; Ren et al. 2012). The sequences that showed 100 % or over 99 % similarity with the sequences analyzed in this study were isolated from different mammals and from immunocompetent or HIV-infected human patients who had clinical symptoms of cryptosporidiosis (diarrhea) and/or the infection was confirmed by microscopy and direct immunofluorescence test (Gatei et al. 2002, 2003; Kváč et al. 2009; Muthusamy et al. 2006; Satoh et al. 2005; Šoba et al. 2006). As the analysis of the sequences obtained from Polish lakes included relatively long fragment of 18S rDNA, it is possible that the strains infective to human and animals were identified. All these sequences clustered together with the sequences representing *C. parvum* bovine genotype, isolated from cattle and human (Fig. 1), and this genotype grouped separately from the clad containing ferret and mouse genotypes of *C. parvum*, what is in accordance with results obtained by others (Šlapeta 2006, 2013; Ren et al. 2012) and supports a suggestion that the bovine genotype should be a separate species.

*G. intestinalis* is a complex species containing eight genotypes that are proposed to be distinct species due to the large genetic distances between them (Ryan and Cacciò 2013). These data are supported by the phylogenetic tree showed in this study (Fig. 2) that consists of two big clades—one containing assemblage B sequences isolated from humans and water, and the other, consisting of assemblage A sequences isolated from humans and other mammals. In the present study, four PCR products of *Giardia* β-giardin sequence were analyzed and the assemblage B of *G. intestinalis* was identified in all samples. In two samples, a sequence identical to 12 *G. intestinalis* sequences from GenBank obtained from humans was identified, whereas in remaining two samples unique sequences were detected and their similarity to the human-originating sequences was over 99 %. The sequences obtained by others from humans were isolated from stool of patients with diarrhea and giardiasis confirmed by different methods including microscopy, fecal antigen test, and immunofluorescence (Cacciò et al. 2002; Guy et al. 2004; Lebbad et al. 2011; Robertson et al. 2007; Abe and Teramoto 2012). It is not possible to determine a subassemblage of the sequences analyzed in this study, because they are much shorter than those obtained by others. On the other hand, it may be difficult even on the basis of multi-locus analysis because different subassemblages sequences of different loci were detected in the same isolates (Cacciò et al. 2002; Robertson et al. 2007).

Four of the five water bodies examined in this study are used for recreational activities (bathing, fishing, and sailing) during summer months, and the water samples were collected from the bathing places, so there is a risk of both cryptosporidiosis and giardiasis in north-western Poland. Although genotyping is crucial in determination of the (oo)cysts origin in water because of *Cryptosporidium* and *Giardia* host specificity, knowing the examined water bodies and their environment are also helpful. *C. parvum* (the bovine genotype) infects mainly human and pre-weaned calves, and dairy calves less than 2 months age are the major contributors of this zoonotic species. However, some epidemiological studies implicated farm (e.g., sheep) and companion (e.g., dogs and horses) animals as a source of human cryptosporidiosis (Hajdušek et al. 2004; Xiao and Fayer 2008; Xiao 2010; Chalmers et al. 2011). What is more, the source of *C. parvum* in human can be also of human origin and many cases of human *C. parvum* infections may be not zoonotic (Xiao and Fayer 2008; Xiao 2010). The host adaptation of *Cryptosporidium* spp. is not strict host specificity and cross-species transmissions may occur (Xiao and Feng 2008), e.g., natural infections with *C. parvum* bovine genotype have been also found in fox, red deer, and rodents (Hajdušek et al. 2004; Lv et al. 2009; Robinson et al. 2011). The host distribution of *G. intestinalis* assemblage B is associated mainly with human and other primates and to a much lesser extent with wildlife (e.g., foxes, wolves, beavers, or few birds species) and dogs (Dado et al. 2012; Ryan and Cacciò 2013). *C. parvum* bovine genotype and *G. intestinalis* assemblage B were detected in five lakes in north-western Poland. Dabie Duze and Dabie Male lakes are the parts of one large water body that adjoins mainly inhabited areas and they are used as sewage discharge places by the local house owners. Glebokie Lake is also a sewage discharge place and is located near a stud farm. Thus, the contamination of the three lakes originates most probably from human, especially in case of *G. intestinalis* assemblage B associated mainly with human. *C. parvum* bovine genotype is associated both with cattle and human; however, there are no cattle farms or fertilized cultivated fields near Glebokie and both Dabie Lakes. Horses may be a minor source of contamination in case of Glebokie Lake; nevertheless, they are hosts rather for the horse genotype of *C. parvum* than the bovine genotype (Xiao and Fayer 2008; Xiao 2010) and the assemblage B of *G. intestinalis* that may be present in horses (Ryan and Cacciò 2013) was not detected in this water body. Weltynskie Lake is surrounded by inhabited and recreational areas, as well as by Puszcza Bukowa forest and serves as a watering place for wild animals. In case of this water body, where assemblage B of *G. intestinalis* was identified, the contamination originates most likely from wildlife. Rusalka Lake is a little water body placed in a public garden near the centre of Szczecin; however, some species of wild animals (e.g., foxes or roe deer) are often observed in the park at night time, while during the day there are many household dogs in this area. Human and cattle are not a probable source of *Cryptosporidium* and *Giardia* (oo)cysts in this water body, thus, wild animals and domestic dogs seem to play this role.

## Conclusions

Only molecular techniques allow detecting infectious *Cryptosporidium* and *Giardia* species and genotypes, so genetic characteristic of these protozoa occurring in water bodies is crucial to evaluate the health risk as well as to determine the contamination source due to the host specificity of these pathogens. However, non-specific relationship between hosts and pathogens may occur and the knowledge of the examined water bodies and their environment is also needed to define the source of contamination. In some cases, minor hosts may be of great importance in spreading *Cryptosporidium* and *Giardia* (oo)cysts in natural water reservoirs. In this study, such hosts seem to play the role of contamination sources of two from five examined lakes where pathogenic genotypes of *C. parvum* and *G. intestinalis* were found.

## References

[CR1] Abe N, Teramoto I (2012). Molecular evidence for person-to-person transmission of a novel subtype in *Giardia duodenalis* assemblage B at the rehabilitation institution for developmentally disabled people. Parasitol Res.

[CR2] Adamska M, Sawczuk M, Kolodziejczyk L, Skotarczak B (2014) Assessment of molecular methods as a tool for detecting of pathogenic protozoa isolated from water tanks, in press 10.2166/wh.2015.077 26608757

[CR3] Cacciò SM, De Giacomo M, Pozio E (2002). Sequence analysis of the β-giardin gene and development of a polymerase chain reaction—Restriction fragment length polymorphism assay to genotype *Giardia duodenalis* cysts from human faecal samples. Int J Parasitol.

[CR4] Chalmers RM, Smith RP, Hadfield SJ, Elwin K, Giles M (2011). Zoonotic linkage and variation in *Cryptosporidium parvum* from patients in the United Kingdom. Parasitol Res.

[CR5] Dado D, Montoya A, Blanco MA, Miró G, Saugar JM, Bailo B, Fuentes I (2012). Prevalence and genotypes of *Giardia duodenalis* from dogs in Spain: Possible zoonotic transmission and public health importance. Parasitol Res.

[CR6] Gatei W, Suputtamongkol Y, Waywa D, Ashford RW, Bailey JW, Greensill J, Beeching NJ, Hart CA (2002). Zoonotic species of *Cryptosporidium* are as prevalent as the anthroponotic in HIV-infected patients in Thailand. Ann Trop Med Parasitol.

[CR7] Gatei W, Greensill J, Ashford RW, Cuevas LE, Parry CM, Cunliffe NA, Beeching NJ, Hart CA (2003). Molecular analysis of the 18S rRNA gene of *Cryptosporidium* parasites from patients with or without human immunodeficiency virus infections living in Kenya, Malawi, Brazil, the United Kingdom, and Vietnam. J Clin Microbiol.

[CR8] Guy RA, Xiao C, Horgen PA (2004). Real-Time PCR assay for detection and genotype differentiation of *Giardia lamblia* in stool specimens. J Clin Microbiol.

[CR9] Hajdušek O, Ditrich O, Šlapeta J (2004). Molecular identification of *Cryptosporidium* spp. in animal and human hosts from the Czech Republic. Vet Parasitol.

[CR10] Kváč M, Květoňová D, Sak B, Ditrich O (2009). *Cryptosporidium* pig genotype II in immunocompetent man. Emerg Infect Dis.

[CR11] Lebbad M, Petersson I, Karlsson L, Botero-Kleiven S, Andersson JO, Svenungsson B, Svärd SG (2011). Multilocus genotyping of human *Giardia* isolates suggests limited zoonotic transmission and association between assemblage B and flatulence in children. PLoS Negl Trop Dis.

[CR12] Lv C, Zhang L, Wang R, Jian F, Zhang S, Ning C, Wang H, Feng C, Wang X, Ren X, Qi M, Xiao L (2009). *Cryptosporidium* spp. in wild, laboratory, and pet rodents in China: Prevalence and molecular characterization. Appl Environ Microbiol.

[CR13] Muthusamy D, Rao SS, Ramani S, Monica B, Banerjee I, Abraham OC, Mathai DC, Primrose B, Muliyil J, Wanke CA, Ward HD, Kang G (2006). Multilocus genotyping of *Cryptosporidium* sp. isolates from human immunodeficiency virus-infected individuals in South India. J Clin Microbiol.

[CR14] Ren X, Zhao J, Zhang L, Ning C, Jian F, Wang R, Lv C, Wang Q, Arrowood MJ, Xiao L (2012). *Cryptosporidium tyzzeri* n. sp. (Apicomplexa: Cryptosporidiiae) in domestic mice (*Mus musculus*). Exp Parasitol.

[CR15] Robertson LJ, Forberg T, Hermansen L, Gjerde BK, Langeland N (2007). Molecular characterisation of *Giardia* isolates from clinical infections following a waterborne outbreak. J Infect.

[CR16] Robinson G, Chalmers RM, Stapleton C, Palmer SR, Watkins J, Francis C, Kay D (2011). A whole water catchment approach to investigating the origin and distribution of *Cryptosporidium* species. J Appl Microbiol.

[CR17] Rossle NF, Latif B (2013). Cryptosporidiosis as threatening health problem: a review. Asian Pac J Trop Biomed.

[CR18] Ruecker NJ, Matsune JC, Wilkes G, Lapen DR, Topp E, Edge TA, Sensen CW, Xiao L, Neumann NF (2012). Molecular and phylogenetic approaches for assessing sources of *Cryptosporidium* contamination in water. Water Res.

[CR19] Ryan U, Cacciò SM (2013). Zoonotic potential of *Giardia*. Int J Parasitol.

[CR20] Satoh M, Kimata I, Iseki M, Nakai Y (2005). Gene analysis of *Cryptosporidium parvum* HNJ-1 strain isolated in Japan. Parasitol Res.

[CR21] Šlapeta J (2006). *Cryptosporidium* species found in cattle: a proposal for new species. Trends Parasitol.

[CR22] Šlapeta J (2013). Cryptosporidiosis and *Cryptosporidium* species in animals and humans: a thirty colour rainbow?. Int J Parasitol.

[CR23] Šoba B, Petrovec M, Mioč V, Logar J (2006). Molecular characterization of *Cryptosporidium* isolates from humans in Slovenia. Clin Microbiol Infect.

[CR24] Tamura K, Peterson D, Peterson N, Stecher G, Nei M, Kumar S (2011). MEGA5: molecular evolutionary genetics analysis using maximum likelihood, evolutionary distance, and maximum parsimony methods. Mol Biol Evol.

[CR25] Xiao L (2010). Molecular epidemiology of cryptosporidiosis: an update. Exp Parasitol.

[CR26] Xiao L, Fayer R (2008). Molecular characterization of species and genotypes of *Cryptosporidium* and *Giardia* and assessment of zoonotic transmission. Int J Parasitol.

[CR27] Xiao L, Feng Y (2008). Zoonotic cryptosporidiosis. FEMS Immunol Med Microbiol.

